# MicroRNA Expression Profiling Altered by Variant Dosage of Radiation Exposure

**DOI:** 10.1155/2014/456323

**Published:** 2014-09-16

**Authors:** Kuei-Fang Lee, Yi-Cheng Chen, Paul Wei-Che Hsu, Ingrid Y. Liu, Lawrence Shih-Hsin Wu

**Affiliations:** ^1^Institute of Medical Sciences, Tzu Chi University, No. 701, Zhongyang Road, Section 3, Hualien 97004, Taiwan; ^2^Laboratory for Cytogenetics, Center for Genetic Counseling, Buddhist Tzu Chi General Hospital, Hualien 97004, Taiwan; ^3^Department of Computer Science & Information Engineering, Tamkang University, New Taipei City 25137, Taiwan; ^4^Bioinformatics Core Laboratory, Institute of Molecular Biology, Academia Sinica, Taipei 11529, Taiwan; ^5^Department of Molecular Biology and Human Genetics, Tzu Chi University, Hualien 97004, Taiwan

## Abstract

Various biological effects are associated with radiation exposure. Irradiated cells may elevate the risk for genetic instability, mutation, and cancer under low levels of radiation exposure, in addition to being able to extend the postradiation side effects in normal tissues. Radiation-induced bystander effect (RIBE) is the focus of rigorous research as it may promote the development of cancer even at low radiation doses. Alterations in the DNA sequence could not explain these biological effects of radiation and it is thought that epigenetics factors may be involved. Indeed, some microRNAs (or miRNAs) have been found to correlate radiation-induced damages and may be potential biomarkers for the various biological effects caused by different levels of radiation exposure. However, the regulatory role that miRNA plays in this aspect remains elusive. In this study, we profiled the expression changes in miRNA under fractionated radiation exposure in human peripheral blood mononuclear cells. By utilizing publicly available microRNA knowledge bases and performing cross validations with our previous gene expression profiling under the same radiation condition, we identified various miRNA-gene interactions specific to different doses of radiation treatment, providing new insights for the molecular underpinnings of radiation injury.

## 1. Introduction

Radiation exists everywhere in our daily life. It is used in medical treatments and also utilized to generate electricity. Risk assessment of acute radiation injury caused by high-dose radiation exposure has been the focus of extensive research, but the mechanisms underlying the effect of low-dose radiation, whether short- or long-term, remain elusive [1]. Response to radiation-induced damages varies due to many confounding factors such as the immune status, age, and genetics [2]. However, in some cases, signs of radiation damage may not be immediately apparent, or not present at all.

Radiation studies primarily concentrate on examining the biological effects of radiation on cell death, chromosomal impairments, mutagenesis, carcinogenesis, and structural alterations of the cell, as well as direct or indirect damage to the DNA double helix via the production of free radical [3]. These damaging consequences of radiation exposure may require several months to years or even generations to develop [4, 5].

In addition to cellular and molecular damages caused by radiation exposure, the radiation-induced bystander effect (RIBE) is also an important topic of rigorous research. The RIBE theory describes the condition in which nonirradiated cells become irradiated by receiving radiation from neighboring irradiated cells. In other words, cells that are not directly hit by an alpha particle but are in the vicinity of one that has been hit also contribute to the genotoxic response of the cell population [6]. RIBE is also suggested to play a role in the biological consequences of exposure to low doses of radiation [7]. Immune cells such as T-lymphocytes and dendritic cells have been particularly implicated in this process [8], though there is currently insufficient evidence to demonstrate that the bystander effect is able to promote carcinogenesis in human at low doses [1]. The mechanisms underlying the bystander effect are complex and a comprehensive understanding of this process has yet to be established [9]. It is known that irradiated cells affect nonirradiated cells through intracellular communications. Molecular signals may be transmitted from the irradiated cells to the nonirradiated ones via gap junctions between cells or through ligand-receptor interaction when the signals are secreted as soluble factors into the culture medium [10]. These transmittable factors are diverse. At present, it is not definitely established as to how many types of molecular signals are involved and to what extent they modulate the transmission of irradiation effect. Nonetheless, RIBE has clear negative implications on health. In the context of RIBE, even at low levels of radiation exposure [11], irradiated cells may still elevate the risk for genetic instability, mutation, and cancer, in addition to being able to extend the postradiation side effects in normal tissues [12]. The bystander effects seem to be tissue-specific as demonstrated by in vivo and in vitro studies [13, 14], and radiation-induced genomic instability (RIGI) as a result of RIBE may play an important role in carcinogenesis [15], though the mechanisms are less clear under fractionated irradiation. RIGI is one of the postirradiation outcomes that appear in nonirradiated progeny cells much later after the initial exposure [15]. Alterations in the DNA sequence could not explain these biological effects of radiation and it is thought that epigenetics, including DNA methylation, histone modifications, chromatin remodeling, and noncoding RNA modulation, may be involved [16].

Indeed, some microRNAs (or miRNAs) were found to be correlated with the biological effects caused by radiation in recent findings. Studies in humans [17, 18] and mice [19, 20] have revealed an association between miRNA regulation and radiation exposure that is dependent on the dosages and time after irradiation. However, the regulatory role miRNA plays in this aspect is still unclear. In the present study, we profiled the expression changes in miRNA under fractionated radiation exposure in human peripheral blood mononuclear cells. By utilizing publicly available microRNA knowledge bases and cross validating with our previous gene expression profiling under the same radiation set-up, our analysis identified specific miRNA-gene interactions characteristic of various doses of radiation treatment, providing new insights for the molecular underpinnings of radiation injury.

## 2. Materials and Methods

### 2.1. Sample Preparation

Whole blood samples (30 mL) were drawn from each of the five participants and collected into vacutainers containing sodium heparin. Samples were irradiated using ^60^Co at a dose rate of 0.546 Gy/min (The Institute of Nuclear Energy Research (INER), Taoyuan, Taiwan). The radiation doses used in these experiments were chosen to cover a range of 0.5 Gy, 1 Gy, 2.5 Gy, and 5 Gy. The control samples were not exposed to any radiation. Samples were harvested after 24 hours of treatment with radiation [21, 22]. Informed consents were obtained from all participants. All procedures were approved by the Institutional Review Board at Tzu Chi General Hospital, Hualien, Taiwan.

### 2.2. RNA Preparation

Total RNA was extracted from peripheral blood mononuclear cells using Trizol (Invitrogen, Carlsbad, CA, USA) according to the manufacturer's instruction. RNA quantity and purity were assessed using NanoDrop ND-1000 (Thermo Fisher Scientific, Waltham, Massachusetts, USA). A260/A280 ≥ 1.6 and A260/A230 ≥ 1 indicate acceptable RNA purity, while acceptable RIN value ≥ 5 using Agilent RNA 6000 Nanoassay (Agilent Technologies, Inc., Santa Clara, California, USA). gDNA contamination was evaluated by gel electrophoresis.

### 2.3. miRNA Expression Profiling

Total RNA samples (2.5 *μ*g) were subjected to microarray analysis of microRNA expression using the Human miRNA OneArray v5 (Phalanx Biotech Group, Hsinchu, Taiwan). Labeling efficiency was calculated by the concentration of CyDye and RNA was measured by NanoDrop ND-1000. Normalization and statistical analysis were conducted with R/Bioconductor (Bioconductor, Fred Hutchinson Cancer Research Center). Expression profiles of changes induced by the various radiation doses (0.5 Gy, 1 Gy, 2.5 Gy, and 5 Gy) were each normalized to the control without any radiation exposure. Significantly differentially expressed miRNAs (normalized intensity ≥ 300, absolute Log_2_ ratio ≥ 1, absolute fold change ≥ 1, and FDR < 0.05) were categorized into up- and downregulated genes for each radiation dose.

### 2.4. miRNA Target Prediction

**Figure 1:**
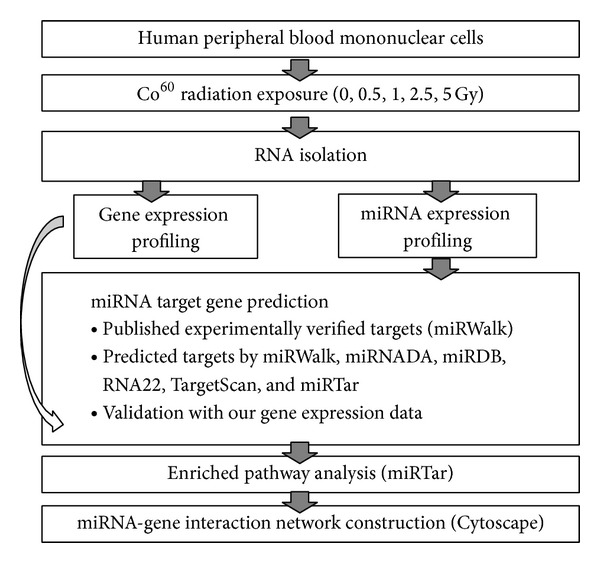
System flow of our analysis.

We adopted an integrative approach, utilizing publicly available databases and our own gene expression data, to identify the target genes for the differentially expressed miRNAs (Figure 1). First, a list of validated targets for a specific differentially expressed miRNA was generated by performing a search through the validated data in miRWalk database [23]. For the predicted data, the accepted target predictions were those identified by at least four out of the five well-established databases, including miRWalk [23], miRANDA [24], miRDB [25], RNA22 [26], and TargetScan [27]. Next, we utilized a list of differentially expressed genes identified from our previous gene expression study of changes in human peripheral blood mononuclear cells induced by varying doses of ^60^Co radiation (absolute Log_2_ ratio ≥ 1, absolute fold change ≥ 1, and FDR < 0.05). For each ^60^Co radiation dosage, we grouped the downregulated genes with upregulated microRNAs and vice versa. On another web-based system, miRTar [28], we input these genes to look for possible miRNA-gene interactions based on the target genes' 3′UTR (untranslated regions), 5′UTR, and coding regions. This systematic approach filtered out previously identified candidate genes that did not match the predicted or validated miRNA-gene interaction list.

### 2.5. miRNA-Gene Interaction Analysis

By employing the gene enrichment function in miRTar [28], the putative or experimentally verified miRNA-gene interactions identified through the integrative database searches were mapped to KEGG (Kyoto Encyclopedia of Genes and Genomes) [29] to reveal the biological importance of these molecular relationships. Only those enriched pathways with a *P* value < 0.05 were considered significant. After applying such a threshold, only miRNAs differentially expressed under 1 Gy of radiation exposure were retained. To visualize the relationships between the candidate miRNAs and their differentially expressed target genes, an interaction network integrating the expression values of each miRNA and gene was constructed using Cytoscape v.3.1.0 (National Institute of General Medical Sciences and National Resource for Network Biology, USA) [30, 31].

## 3. Results

Few miRNAs showed significant changes in expression under 0.5 and 2.5 Gy of ^60^Co radiation exposure (Table 1). Whereas only four miRNAs exhibited enhanced expression under 5 Gy of radiation, a dosage of 1 Gy appeared to induce expression changes in the greatest number of microRNAs, with seven being upregulated and two downregulated. The pattern is consistent with our findings [32] on the gene expression changes in human peripheral blood mononuclear cells exposed to the same varying doses of ^60^Co radiation.

To elucidate the potential regulatory relationship between the differentially expressed miRNAs and our previously identified gene candidates [32], we performed a systematic search utilizing a variety of database tools.  Table 2 presents the potential relationships between the candidate miRNAs and their corresponding target genes as validated by our list of differentially expressed genes. It appears that most of the molecular changes occurred at 1 Gy of radiation dosage.

In order to identify the biological importance of the molecular changes occurring under 1 Gy of radiation exposure, the differentially expressed miRNAs and their corresponding target genes were mapped to their respective KEGG pathways. The overrepresented pathways seem to be related to homeostasis, metabolism, neuronal survival, and cellular control (Table 3). Three microRNAs, hsa-miR-20b-5p, hsa-miR-17-5p, and hsa-miR-185-5p, appear to regulate the highest number of radiation sensitive genes compared to the other differentially expressed microRNAs (Table 2).

**Figure 2:**
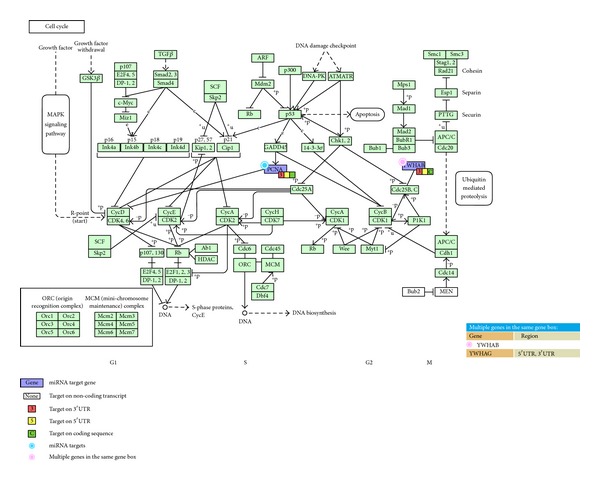
Regulation of* YWHAG*,* YWHAB*, and* PCNA* by hsa-miR-185-5p in a cell cycle pathway.

**Figure 3:**
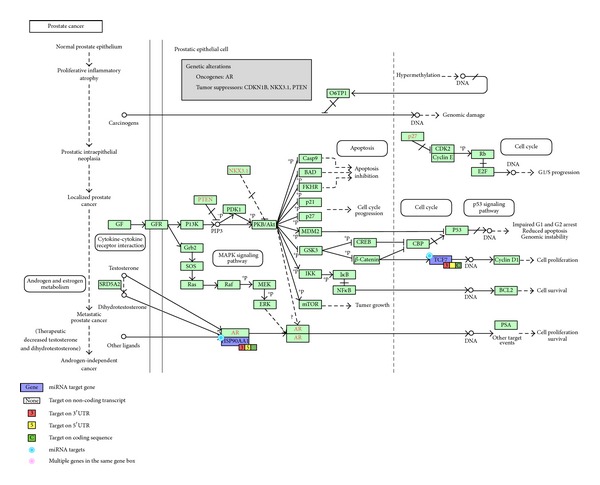
Regulation of* TCF7* and* HSP90AA1* by hsa-miR-185-5p in a prostate cancer pathway.

**Figure 4:**
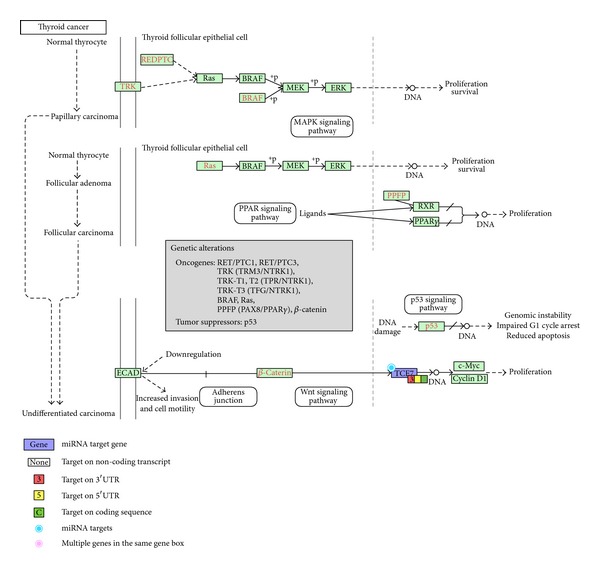
Regulation of* TCF7* by hsa-miR-20b-5p and hsa-miR-17-5p in a thyroid cancer pathway.

Moreover, these miRNAs' target genes are enriched in cancer and cell cycle-related pathways (Table 3). Consequently, hsa-miR-20b-5p, hsa-miR-17-5p, and hsa-miR-185-5p may be involved in modulating genes underlying cell cycle control and the development of thyroid cancer and prostate cancer. The pathway-specific association between these miRNAs and their corresponding target genes is shown in Figures 2–4.

**Figure 5:**
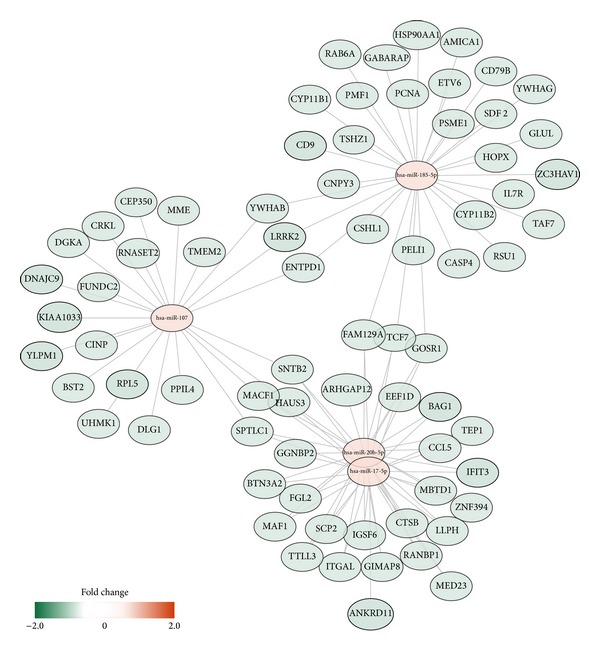
Potential miRNA-gene interaction network associated with the changes induced by 1 Gy of ^60^Co radiation exposure in human peripheral blood mononuclear cells.

Our data suggest that the miRNA-gene interactions associated with 1 Gy of radiation dosage treatment may be the key molecular signatures underlying the damages caused by radiation exposure. In order to visualize the relationships between these miRNA and gene candidates, we constructed an interaction network that illustrates the complex regulatory relationships among these genes and miRNAs. Note that hsa-miR-20b-5p and hsa-miR-17-5p share many target genes, suggesting that they modulate gene expression through a cooperative manner (Figure 5).

## 4. Discussion

In the current study, we profiled miRNA expression changes under varying doses of radiation exposure through an array-based approach. Our results support the emerging evidence that tissue and cellular injuries may alter miRNA expression [33]. In addition, by utilizing publicly available bioinformatics resources and comparing with our previous gene expression profiling data, we have mapped out a potential miRNA-gene interactome map that may underlie the molecular changes induced by radiation treatment.

Most of the upregulated miRNAs were found under low doses of ^60^Co radiation exposure (≤1 Gy), while only significantly downregulated miRNAs were found in the 5 Gy radiation dosage range. This indicates that response to radiation exposure at the miRNA level is dose dependent. From publicly available miRNA knowledge bases, we retrieved a list of genes that have been validated to interact with these miRNAs, namely, hsa-miR-185-5p, hsa-miR-107, hsa-miR-20b-5p, and hsa-miR-17-5p for the low radiation doses and hsa-miR-142, hsa-miR-223-3p, and hsa-miR-451a for the 5 Gy radiation exposure. These genes were compared to those identified from our previous gene expression profiling experiment. Matched genes were considered the most promising targets.

Our analysis showed that two miRNAs, hsa-miR-142-3p and hsa-miR-223-3p, with decreased expression after exposure to 5 Gy of ^60^Co radiation may be potential modulators of* MAP4K3* (mitogen-activated protein kinase kinase kinase kinase 3) and* DUSP10* (dual specificity phosphatase 10), respectively. In particular, MAP4K3 is an apoptosis inducer that is activated upon UV radiation [34]. Mutation in the* MAP4K3* gene sequence was predicted to modulate cancer progression [35], and this hypothesis was supported by abnormal levels of MAP4K3 expression in pancreatic cancer tissues and enhanced cellular proliferation by RNAi-induced suppression of* MAP4K3* [36]. On the other hand, increased transcript abundance of DUSP10 was shown to influence gut homeostasis by suppressing proliferation and apoptosis, while promoting differentiation [37]. Both DUSP10 and MAP4K3 play important roles in the MAPK signaling pathway [38]. Our results suggest that the increase in* MAP4K3* and* DUSP10* transcript abundance as a result of 5 Gy radiation exposure may be modulated by their interactions with hsa-miR-142-3p and hsa-miR-223-3p, respectively. These putative relationships might even be one of the underlying regulatory mechanisms of radiation-induced changes in cell cycle signaling.

As the upregulated miRNAs were primarily found in the low-dose radiation range (0.5 Gy and 1 Gy), we performed a series of gene set enrichment analysis on their candidate target genes and mapped these miRNA-gene interactions to pathways related to the cell cycle, neurotrophin signaling, renin-angiotensin system, and pentose phosphate pathways, as well as prostate and thyroid cancers. This is in line with the observation that radiation induced apoptosis through the neurotrophin signaling pathway [39]. Delayed wound healing following low-dose radiation exposure was also partially attributed to reduced activity of the renin-angiotensin system [40]. Moreover, evidence has shown that increased activity of the pentose phosphate cycle can protect cells from programmed cell death induced by low doses of ionizing radiation [41]. According to our results, it is possible that specific miRNAs are upregulated to modulate genes involved in these pathways in response to low-dose radiation.

The miRNA hsa-miR-185-5p exhibited increased expression when exposed to 0.5 Gy and 1 Gy dosage of radiation and was predicted to interact with* YWHAG* (tyrosine 3-monooxygenase/tryptophan 5-monooxygenase activation protein, gamma polypeptide),* YWHAB* (tyrosine 3-monooxygenase/tryptophan 5-monooxygenase activation protein, beta polypeptide), and* PCNA* (proliferating cell nuclear antigen), which appeared to be downregulated under the same condition. Pathway analysis (see Figure 2) suggested that these three genes are involved in cell cycle pathways. In particular, YWHAB is known to regulate the cyclin B and cyclin-dependent kinase 1 complex, while PCNA is an inhibitor of the cyclin D and cyclin-dependent kinase 4 or 6 complex [42, 43]. According to a porcine model study, YWHAG and YWHAB mediate insulin-like growth factor signaling and the G2/M DNA damage checkpoint in cell cycle control [44]. Maternal high protein diet has been shown to associate with increasing expression levels of* YWHAG* and* YWHAB* [44]. Our results support that these three genes regulate the cell cycle pathway and are particularly sensitive to changes in the external environment, especially radiation exposure, and that hsa-miR-185-5p may be involved in mediating this response. In addition, we showed that hsa-miR-185 may control the expression of* TCF7* (T-cell-specific transcription factor 7) and* HSP90AA1* (heat shock protein 90 kDa alpha, class A member 1) in Figure 3. TCF7 is a known regulator of the Wnt signaling pathway [45] and an important modulator of the self-renewal and differentiation processes in hematopoietic cells [46]. In contrast,* HSP90AA1* is responsible for the degradation of androgen receptor and cell killing following radiation exposure in a prostate cancer cell line [47]. In addition, hsa-miR-185 is also known to be involved in the development of prostate cancer [48]. Our pathway analysis further supports the roles these three molecules play in carcinogenesis by mapping the interaction among hsa-miR185-5p,* TCF7*, and* HSP90AA1* to prostate cancer through the PI3K signaling pathway.

Previous study reported that hsa-miR-107 regulates the DNA damage response (DDR) and sensitizes tumor cells by repressing expression of* RAD51* and corporation with miR-222 in olaparib, an experimental chemotherapeutic agent, thus impairing DSB repair by HR [49]. Elevated expression of miR-107 has been correlated with* PARP* inhibitor sensitivity and reduced* RAD51* expression in a subset of ovarian clear cell carcinomas [49]. The miRNAs hsa-mir-103 and hsa-mir-107 are upregulated in relation to insulin sensitivity in an obese mouse model. This suggested that these miRNAs represent potential biomarkers for type 2 diabetes (the miR-103 microRNA precursor is homologous to miR-107) [50]. The miR-107 negatively regulates the miRNA let-7 via direct interaction in tumors and in cancer cell line. Previous study showed that miR-107 increased the tumourigenic and metastatic potential via inhibition of let-7 and upregulation of let-7 targets in human breast cancer cell line and in mice model [51]. Our result indicates that miR-107 is upregulated under 1 Gy dosage of radiation exposure and this increase in expression is associated with metabolic pathways and potentially involved in cancer development. Three genes,* MME*,* YWHAB*, and* CRKL*, were predicted to interact with miR-107.* MME* was involved in rennin-angiotensin pathway and* CRKL* and* YWHAB* were involved in neurotrophin signaling pathway in our data. Our analysis demonstrated that miR-107 may cooperate with miR-185-5p to regulate the cell cycle via* YWHAB*. This result corresponds to a previous study, which showed that the miR-107 and miR-185, localized in frequently altered chromosomal regions in human lung cancers, may contribute to regulate cell cycle in human malignant tumors [52].

In addition, our analysis in Figure 4 suggests that miR-17-5p and miR-20b-5p may cooperate to exert regulatory effects on the* TCF7* gene. In fact, miR-17-5p and miR-20b-5p are mature forms of the same precursor family. The microRNA miR-17-5p has been shown to mediate the transition from G1 to S phase of the cell cycle and initiate the signal for proliferation [53, 54]. Moreover, miR-17-5p can act as both an oncogene and a tumor suppressor gene in different cellular contexts, underscoring its importance in cell cycle control [53]. Our finding indicates that miR-17-5p and miR-20b-5p may modulate the Wnt signaling pathway by regulating* TCF7* expression, which in turn affects the activity of the c-Myc and cyclin D1 complex. This particular process is associated with thyroid cancer. Thus, under low-dose radiation, changes in the abundance of miR-17-5p and miR-20b-5p may influence the cell cycle via interaction with their target gene* TCF7* and modulate the development of thyroid cancer [55, 56].

Our study demonstrates that many miRNAs were upregulated in response to low-dose radiation, and these radiation-induced changes may alter cell cycle regulation, affecting cell rescue, interrupt the generation of NADPH and pentoses through glycolysis, create imbalance in body fluid homeostasis via the renin-angiotensin system (RAS), and finally modulate cell survival through the neurotrophin signaling. In conclusion, we have provided comprehensive miRNA-gene interaction networks that underlie the mechanisms of damages induced by varying doses of radiation. Our findings have built a framework for further validation studies to investigate the specific molecular signatures of radiation exposure.

## Supplementary Material

miRNA-gene interaction predicted by miRTar; the complete gene list is provided by our array original data.

## Figures and Tables

**Table 1:** Number of significantly differentially expressed miRNAs in human peripheral blood mononuclear cells exposed to varying doses of ^60^Co radiation (absolute fold change ≥ 1; FDR < 0.05).

Comparison (Gy)	Upregulated miRNAs	Downregulated miRNAs
0 versus 0.5	hsa-miR-185-5p	0

0 versus 1	hsa-miR-107	hsa-miR-3180
	hsa-miR-126-3p	hsa-miR-4730
	hsa-miR-144-3p	
	hsa-miR-17-5p	
	hsa-miR-185-5p	
	hsa-miR-20b-5p	
	hsa-miR-5194	

0 versus 2.5	0	0

0 versus 5	0	hsa-miR-142-3p
		hsa-miR-142-5p
		hsa-miR-223-3p
		hsa-miR-451a

**Table 2:** Putative and validated interactions between the differentially expressed microRNAs and gene candidates specific to each dose of ^60^Co radiation.

Dose (Gy)	miRNA	Fold change	Target gene	Fold change
0.5	hsa-miR-185-5p	1.18	*TNFSF10* ^ a^	−1.21
			*GLUL* ^ c^	−1.49

1	hsa-miR-107	1.09	14 genes^c^	Decreased
	hsa-miR-144	1.60	*SELL *	−1.04
			*COTL *	−1.06
			*CEP63 *	−1.14
	hsa-miR-17-5p	1.09	*MCL1* ^ a^	−1.26
			*FGL2* ^ b^	−1.33
			30 other genes^c^	Decreased
	hsa-miR-185-5p	1.01	32 genes^c^	Decreased
	hsa-miR-20b-5p	1.01	27 genes^c^	Decreased

5	hsa-miR-142-3p	−1.02	*MAP4K3* ^ b^	1.32
			*DIRC2* ^ b^	1.01
			*TIPARP* ^ b^	1.05
			*PDE4B* ^ c^	1.54
	hsa-miR-142-5p	−1.09	*AHR* ^ a^	1.31
			*SLC36A4* ^ b^	1.11
	hsa-miR-223-3p	−1.27	*DUSP10* ^ b,c^	1.07
			*EFNA1* ^ b^	1.01
	hsa-miR-451a	−1.28	*SLC7A11* ^ c^	1.14

^a^Experimentally validated miRNA-gene interaction as identified by miRWalk.

^b^miRNA-gene interaction predicted by at least four out of the five selected miRNA target prediction databases.

^c^miRNA-gene interaction predicted by miRTar; the complete gene list is provided in Supplementary Material 1 (see Supplementary Material available online at http://dx.doi.org/10.1155/2014/456323).

**Table 3:** Enriched KEGG pathways associated with specific miRNA-gene interactions under 1 Gy of ^60^Co radiation exposure.

microRNA	Pathway	Genes	*P* value
hsa-miR-185-5p	Cell cycle	*YWHAG, YWHAB,* and* PCNA *	0.0019
	Prostate cancer	*TCF7, HSP90AA1 *	0.0380
hsa-miR-107	Neurotrophin signaling pathway	*YWHAB, CRKL *	0.0200
	Renin-angiotensin system	*MME *	0.0351
hsa-miR-20b-5p	Thyroid cancer	*TCF7 *	0.0490
hsa-miR-17-5p	Pentose phosphate pathway	*TALDO1 *	0.0490
	Thyroid cancer	*TCF7 *	0.0483
