# Supplementary material for: MicroRNA Expression Profiling Altered by Variant Dosage of Radiation Exposure
Source: Biomed Res Int. 2014 Sep 16;2014:456323. doi: 10.1155/2014/456323 (PMC4182081; doi:10.1155/2014/456323)
Supplement: Supplementary file 1 — miRNA-gene interaction predicted by miRTar; the complete gene list is provided by our array original data. [file 456323.f1.pdf]

Supplementary material 1. mirtar prediction

microRNA	microRNA_fold_change	gene_symbols	gene_fold_change	binding
hsa-miR-107	1.085757	BST2	-1.01332	YES
hsa-miR-107	1.085757	CEP350	-1.0824	YES
hsa-miR-107	1.085757	CINP	-1.196687	YES
hsa-miR-107	1.085757	CRKL	-1.228981	YES
hsa-miR-107	1.085757	DGKA	-1.265459	YES
hsa-miR-107	1.085757	DLG1	-1.124829	YES
hsa-miR-107	1.085757	DNAJC9	-1.849915	YES
hsa-miR-107	1.085757	ENTPD1	-1.022713	YES
hsa-miR-107	1.085757	FUNDC2	-1.114613	YES
hsa-miR-107	1.085757	HAUS3	-1.108768	YES
hsa-miR-107	1.085757	KIAA1033	-1.383965	YES
hsa-miR-107	1.085757	LRRK2	-1.022049	YES
hsa-miR-107	1.085757	MACF1	-1.053049	YES
hsa-miR-107	1.085757	MME	-1.346039	YES
hsa-miR-107	1.085757	PPIL4	-1.031364	YES
hsa-miR-107	1.085757	RNASET2	-1.044624	YES
hsa-miR-107	1.085757	RPL5	-1.434504	YES
hsa-miR-107	1.085757	SNTB2	-1.070127	YES
hsa-miR-107	1.085757	SPTLC1	-1.18119	YES
hsa-miR-107	1.085757	TMEM2	-1.050364	YES
hsa-miR-107	1.085757	UHMK1	-1.00323	YES
hsa-miR-107	1.085757	YLPM1	-1.431853	YES
hsa-miR-107	1.085757	YWHAB	-1.146669	YES
hsa-miR-20b-5p	1.010934	ARHGAP12	-1.28223	YES
hsa-miR-20b-5p	1.010934	BAG1	-1.480575	YES
hsa-miR-20b-5p	1.010934	BTN3A2	-1.216127	YES
hsa-miR-20b-5p	1.010934	CCL5	-1.103018	YES
hsa-miR-20b-5p	1.010934	CTSB	-1.02047	YES
hsa-miR-20b-5p	1.010934	EEF1D	-1.037083	YES
hsa-miR-20b-5p	1.010934	FAM129A	-1.000626	YES
hsa-miR-20b-5p	1.010934	FGL2	-1.328617	YES
hsa-miR-20b-5p	1.010934	GGNBP2	-1.114713	YES
hsa-miR-20b-5p	1.010934	GIMAP8	-1.105476	YES
hsa-miR-20b-5p	1.010934	GOSR1	-1.128264	YES
hsa-miR-20b-5p	1.010934	HAUS3	-1.108768	YES

hsa-miR-20b-5p	1.010934	IFIT3	-1.011254	YES
hsa-miR-20b-5p	1.010934	IGSF6	-1.269933	YES
hsa-miR-20b-5p	1.010934	ITGAL	-1.06241	YES
hsa-miR-20b-5p	1.010934	LLPH	-1.027615	YES
hsa-miR-20b-5p	1.010934	MACF1	-1.053049	YES
hsa-miR-20b-5p	1.010934	MAF1	-1.127237	YES
hsa-miR-20b-5p	1.010934	MBTD1	-1.038898	YES
hsa-miR-20b-5p	1.010934	RANBP1	-1.327268	YES
hsa-miR-20b-5p	1.010934	SCP2	-1.254943	YES
hsa-miR-20b-5p	1.010934	SNTB2	-1.070127	YES
hsa-miR-20b-5p	1.010934	SPTLC1	-1.18119	YES
hsa-miR-20b-5p	1.010934	TCF7	-1.207391	YES
hsa-miR-20b-5p	1.010934	TEP1	-1.182007	YES
hsa-miR-20b-5p	1.010934	TTLL3	-1.123415	YES
hsa-miR-20b-5p	1.010934	ZNF394	-1.108821	YES
hsa-miR-17-5p	1.094021	ANKRD11	-1.597491	YES
hsa-miR-17-5p	1.094021	ARHGAP12	-1.28223	YES
hsa-miR-17-5p	1.094021	BAG1	-1.480575	YES
hsa-miR-17-5p	1.094021	BTN3A2	-1.216127	YES
hsa-miR-17-5p	1.094021	CCL5	-1.103018	YES
hsa-miR-17-5p	1.094021	CTSB	-1.02047	YES
hsa-miR-17-5p	1.094021	EEF1D	-1.037083	YES
hsa-miR-17-5p	1.094021	FAM129A	-1.000626	YES
hsa-miR-17-5p	1.094021	FGL2	-1.328617	YES
hsa-miR-17-5p	1.094021	GGNBP2	-1.114713	YES
hsa-miR-17-5p	1.094021	GIMAP8	-1.105476	YES
hsa-miR-17-5p	1.094021	GOSR1	-1.128264	YES
hsa-miR-17-5p	1.094021	HAUS3	-1.108768	YES
hsa-miR-17-5p	1.094021	IFIT3	-1.011254	YES
hsa-miR-17-5p	1.094021	IGSF6	-1.269933	YES
hsa-miR-17-5p	1.094021	ITGAL	-1.06241	YES
hsa-miR-17-5p	1.094021	LLPH	-1.027615	YES
hsa-miR-17-5p	1.094021	MACF1	-1.053049	YES
hsa-miR-17-5p	1.094021	MAF1	-1.127237	YES
hsa-miR-17-5p	1.094021	MBTD1	-1.038898	YES
hsa-miR-17-5p	1.094021	MED23	-1.149971	YES
hsa-miR-17-5p	1.094021	RANBP1	-1.327268	YES
hsa-miR-17-5p	1.094021	SCP2	-1.254943	YES

hsa-miR-17-5p	1.094021	SNTB2	-1.070127	YES
hsa-miR-17-5p	1.094021	SPTLC1	-1.18119	YES
hsa-miR-17-5p	1.094021	TCF7	-1.207391	YES
hsa-miR-17-5p	1.094021	TEP1	-1.182007	YES
hsa-miR-17-5p	1.094021	TTLL3	-1.123415	YES
hsa-miR-17-5p	1.094021	ZNF394	-1.108821	YES
hsa-miR-185-5p	1.012046	AMICA1	-1.021901	YES
hsa-miR-185-5p	1.012046	CASP4	-1.417635	YES
hsa-miR-185-5p	1.012046	CD79B	-1.213594	YES
hsa-miR-185-5p	1.012046	CD9	-1.461641	YES
hsa-miR-185-5p	1.012046	CNPY3	-1.043983	YES
hsa-miR-185-5p	1.012046	CSHL1	-1.230325	YES
hsa-miR-185-5p	1.012046	CYP11B1	-1.123859	YES
hsa-miR-185-5p	1.012046	CYP11B2	-1.123859	YES
hsa-miR-185-5p	1.012046	ENTPD1	-1.022713	YES
hsa-miR-185-5p	1.012046	ETV6	-1.445229	YES
hsa-miR-185-5p	1.012046	FAM129A	-1.000626	YES
hsa-miR-185-5p	1.012046	GABARAP	-1.017526	YES
hsa-miR-185-5p	1.012046	GLUL	-1.454511	YES
hsa-miR-185-5p	1.012046	GOSR1	-1.128264	YES
hsa-miR-185-5p	1.012046	HOPX	-1.15993	YES
hsa-miR-185-5p	1.012046	HSP90AA1	-1.405428	YES
hsa-miR-185-5p	1.012046	IL7R	-1.202742	YES
hsa-miR-185-5p	1.012046	LRRK2	-1.022049	YES
hsa-miR-185-5p	1.012046	PCNA	-1.011372	YES
hsa-miR-185-5p	1.012046	PELI1	-1.162732	YES
hsa-miR-185-5p	1.012046	PMF1	-1.033985	YES
hsa-miR-185-5p	1.012046	PSME1	-1.283355	YES
hsa-miR-185-5p	1.012046	RAB6A	-1.04013	YES
hsa-miR-185-5p	1.012046	RSU1	-1.061515	YES
hsa-miR-185-5p	1.012046	SDF2	-1.245329	YES
hsa-miR-185-5p	1.012046	TAF7	-1.298485	YES
hsa-miR-185-5p	1.012046	TCF7	-1.207391	YES
hsa-miR-185-5p	1.012046	TSHZ1	-1.006175	YES
hsa-miR-185-5p	1.012046	YWHAB	-1.146669	YES
hsa-miR-185-5p	1.012046	YWHAG	-1.161326	YES
hsa-miR-185-5p	1.012046	ZC3HAV1	-1.860208	YES
